# Radiological Findings Increased the Successful of COVID-19 Diagnosis in Hospitalized Patients Suspected of Respiratory Viral Infection but with a Negative First SARS-CoV-2 RT-PCR Result

**DOI:** 10.3390/diagnostics12030687

**Published:** 2022-03-11

**Authors:** Margarita L Martinez-Fierro, Carolina González-Fuentes, Dagoberto Cid-Guerrero, Samantha González Delgado, Santiago Carrillo-Martínez, Edgar Fernando Gutierrez-Vela, Juan Yadid Calzada-Luévano, Maria R. Rocha-Pizaña, Jacqueline Martínez-Rendón, Maria E. Castañeda-López, Idalia Garza-Veloz

**Affiliations:** 1 Molecular Medicine Laboratory, Unidad Académica de Medicina Humana y Ciencias de la Salud, Universidad Autónoma de Zacatecas, Zacatecas 98160, Mexico; carolina.glz.fuentes@gmail.com (C.G.-F.); samanthagonzalez407@gmail.com (S.G.D.); santtchago@gmail.com (S.C.-M.); juan_yadid@hotmail.com (J.Y.C.-L.); jamare85@gmail.com (J.M.-R.); maru.casta77@gmail.com (M.E.C.-L.); 2 Hospital General “Luz González Cosío”, Circuito Ciudad Gobierno, Zacatecas 98160, Mexico; cidgrod@yahoo.com.mx (D.C.-G.); fer_drgtz@yahoo.com.mx (E.F.G.-V.); 3 Escuela de Ingenieria y Ciencias, Tecnologico de Monterrey Campus Puebla, Puebla 72453, Mexico; mrochap@tec.mx

**Keywords:** COVID-19, SARS-CoV-2, RT-PCR, false negative, viral screening, CO-RADS

## Abstract

SARS-CoV-2 is the etiological agent of COVID-19 and may evolve from asymptomatic disease to fatal outcomes. Real-time reverse-transcription polymerase chain reaction (RT-PCR) screening is the gold standard to diagnose severe accurate respiratory syndrome coronavirus 2 (SARS-CoV-2) infection, but this test is not 100% accurate, as false negatives can occur. We aimed to evaluate the potential false-negative results in hospitalized patients suspected of viral respiratory disease but with a negative previous SARS-CoV-2 RT-PCR and analyze variables that may increase the success of COVID-19 diagnosis in this group of patients. A total of 55 hospitalized patients suspected of viral respiratory disease but with a previous negative RT-PCR result for SARS-CoV-2 were included. All the participants had clinical findings related to COVID-19 and underwent a second SARS-CoV-2 RT-PCR. Chest-computed axial tomography (CT) was used as an auxiliary tool for COVID-19 diagnosis. After the second test, 36 patients (65.5%) were positive for SARS-CoV-2 (COVID-19 group), and 19 patients (34.5%) were negative (controls). There were differences between the groups in the platelet count and the levels of D-dimer, procalcitonin, and glucose (*p* < 0.05). Chest CT scans categorized as COVID-19 Reporting and Data System 5 (CO-RADS 5) were more frequent in the COVID-19 group than in the control group (91.7% vs. 52.6%; *p* = 0.003). CO-RADS 5 remained an independent predictor of COVID-19 diagnosis in a second SARS-CoV-2 screening (*p* = 0.013; odds ratio = 7.0, 95% confidence interval 1.5–32.7). In conclusion, chest CT classified as CO-RADS 5 was an independent predictor of a positive second SARS-CoV-2 RT-PCR, increasing the odds of COVID-19 diagnosis by seven times. Based on our results, in hospitalized patients with a chest CT classified as CO-RADS 5, a second SARS-CoV-2 RT-PCR test should be mandatory when the first one is negative. This approach could increase SARS-CoV-2 detection up to 65% and could allow for isolation and treatment, thus improving the patient outcome and avoiding further contagion.

## 1. Introduction

Severe accurate respiratory syndrome coronavirus 2 (SARS-CoV-2) is an RNA virus belonging to the family of SARS-CoV and the Middle East respiratory syndrome coronavirus (MERS-CoV) [1]. SARS-CoV-2 was identified in January 2020 as the etiological agent of an atypical pneumonic epidemic that started in Wuhan, China [1]. This illness was later called coronavirus disease 2019 (COVID-19) and was declared a pandemic by the World Health Organization (WHO) in March 2020 [1]. Up to 26 December 2021, there had been 278 million cases and 5.4 million deaths [2,3]. In Mexico, up to 2 January 2022, there had been 3,990,587 confirmed cases and 299,544 accumulated deaths [4,5].

The SARS-CoV-2 genome encodes multiple non-structural proteins and four structural proteins: spike (S), envelope (E), membrane (M), and nucleocapsid (N). Infection starts with the transmission of aerosols with SARS-CoV-2 which binds to the epithelial nasal cells in the upper respiratory tract of the host [6]. SARS-CoV-2 binds to the cell membrane through the S protein [7] via angiotensin converting enzyme 2 (ACE2) and activates the protease TMPRSS2. After virus entry into the host cell, the viral genome is replicated via a replicase–protein complex encoded by the 20 kilobase (kb) replicase gene comprising two large open reading frames, designated ORF1a and ORF1b [3]. RNA-dependent RNA polymerase (RdRp) and helicase are encoded by ORF1b. The replicase complex includes both cellular proteins and up to 16 viral subunits [3]. After replication, this complex binds to the cell-host membrane and the genomic RNA is incorporated for the assembly of new virus particles, which are released via exocytosis to infect more cells [8]. The virus goes through replication and local propagation with infection of the ciliated cells in the airway. The immune response is limited in this phase, but individuals are highly infectious [6].

There has been a spectrum of COVID-19 severity reported in the literature [9]. While patients with mild COVID-19 can present fever, sore throat, cough without expectoration, general discomfort, nausea, vomiting, and abdominal pain, patients with moderate infection can present pneumonia with persistent fever and cough, but without hypoxemia [9]. Severe infections are characterized by the presence of hypoxemia with pneumonia; oxygen saturation < 92%; and a critical state that corresponds to acute respiratory distress syndrome, coagulation alterations, encephalopathy, cardiac failure, and acute kidney injury [6]. SARS-CoV-2 induces a multisystemic disease. The cytokine storm associated with COVID-19 involves an increase in proinflammatory factors, such as procalcitonin and cytokines [10,11]. The infection also causes a prothrombotic state, and, consequently, patients can present increased levels of reactive C protein, D-dimer, platelets, and fibrinogen [12]. Measuring the levels of these parameters could be used to predict disease severity [10].

COVID-19 diagnosis can be realized through molecular detection of SARS-CoV-2 antigens or RNA. The gold standard for SARS-CoV-2 detection is real-time reverse-transcription polymerase chain reaction (RT-PCR), which uses RNA extracted preferably from nasopharynx and oropharynx exudates [13]. In Mexico, an official national health network of Viral Respiratory Disease Monitoring Health Units is distributed throughout the country; the purpose is epidemiological surveillance. These health units have good laboratory facilities and qualified staff, and since the beginning of COVID-19 pandemic, they have been used to collect and report data about the disease [14]. All the institutions in the national network use the same standardized RT-PCR test based on the Berlin protocol, which detects the E and RdRp genes [15,16]. The E gene is used for screening to detect any beta-coronavirus associated with bats, while the RdRp gene is specific for SARS and SARS-like coronaviruses (including SARS-CoV-2) [15,16]. Other protocols have also been implemented, the most popular of which are to detect the N, E, or S genes and/or ORF1 of SARS-CoV-2 [15]. Nevertheless, a high number of false negatives for SARS-CoV-2 is one of the main challenges for the COVID-19 diagnosis. Indeed, while the accuracy of the RT-PCR method is 80–100%, the false-negative rate is highly variable, between 9.3% and 67% [3,17]. There could be several causes for the high number of false negatives: the method of detection, changes in the sample storage temperature or how long samples are stored, inadequate viral material, laboratory mistakes, and limitations in sample transportation [18].

Researchers have assessed diagnostic tools for COVID-19; the nasopharyngeal swab appears to have higher positivity rates than other samples, especially later in the course of the illness [19]. The evaluation of commercial RT-PCR kits for SARS-CoV-2 has allowed researchers to calculate a sensitivity between 82% and 100%, a specificity of 100% [20], and a precision near 59% [21]. Moreover, the use of alternative samples has been suggested to maintain appropriate precautions in patients suspected of having COVID-19 who had a negative RT-PCR result [22]. Of note, up to 93% of patients with an initial negative test but with a chest-computed axial tomography (CT) scan highly suggestive of COVID-19 had a positive second SARS-CoV-2 RT-PCR result, on average, 5.1 days after the first test [21], highlighting the necessity of a correct COVID-19 diagnosis that will have an impact on the prognosis, in the selection of the pharmacological therapy and clinical interventions, and, therefore, on the patient outcome [13]. In this study, we aimed to evaluate the potential false-negative results in hospitalized patients suspected of viral respiratory disease but with a negative previous SARS-CoV-2 RT-PCR. We also evaluated biochemical, clinical, and imaging variables that may increase the success of COVID-19 diagnosis in this group of patients.

## 2. Materials and Methods

### 2.1. Study Population

This case-control study was carried out from November 2020 to May 2021 and was approved by the Ethic and Investigation Committee of the General Hospital of Zacatecas Luz González Cosío (HGZ) in Zacatecas, Mexico (Protocol ID: 0227/2021/C). Patients who authorized their participation in this protocol by signing the informed consent were included in the study. The inclusion criteria were patients older than 18 years who met the hospitalization criteria according to standards established by the Ministry of Health of Zacatecas [23]. These patients were hospitalized in the area of adults with COVID-19 in the HGZ. Participants had clinical and/or radiological findings suggestive of COVID-19 and had had an initial negative SARS-CoV-2 RT-PCR test. Patients who did not agree to participate in the study and/or who had psychiatric illness were not included. All patients included in the study underwent a second nasopharynx and oropharynx swabs for RT-PCR test for SARS-CoV-2. Based on the result, they were divided into the COVID-19 group (positive SARS-CoV-2 RT-PCR) or the control group (negative SARS-CoV-2 RT-PCR). The COVID-19 group included 36 patients, and the control group included 19 participants.

### 2.2. Clinical and Biochemical Data at the Hospital Admission

The presence of comorbidities in each participant was obtained from their medical record. The clinical manifestations in these patients at the time of hospitalization considered for this study were the presence/absence of symptoms, such as non-productive cough, dyspnea, thoracic pain, fever, anosmia, dysgeusia, asthenia, arthralgias, diarrhea, headache, and nausea. Biochemical test results, including hemoglobin, platelet count, leucocytes, lymphocytes, neutrophils, prothrombin, activated thromboplastin time, glucose, creatinine, ureic nitrogen, direct bilirubin, and albumin, were obtained at the time of admission. Inflammatory markers considered for the study were fibrinogen, D-dimer, lactate dehydrogenase, ferritin, procalcitonin, C-reactive protein, troponin, and creatine kinase (Ck). The need for oxygen supplementation was also recorded.

### 2.3. Clinical Criteria Used to Classify of Patients According to COVID-19 Severity

For each patient, COVID-19 severity was established according to the Mexican Inter-Institutional consensus “Clinical guide for COVID-19 treatment in Mexico” created by the Mexican Government together with Mexican Health authorities [9]. According to this guideline, mild SARS-CoV-2 infection included patients with no evidence of pneumonia or hypoxia (oxygen saturation >94%); moderate infection was considered in cases with clinical evidence of pneumonia (oxygen saturation >90%); and severe infection included all cases with clinical evidence of pneumonia and one of the following conditions, namely respiratory frequency up to 30 breathes per minute, severe respiratory difficulty, and/or oxygen saturation <90%.

### 2.4. Chest CT Scan

At hospital admission, patients with suspicion of COVID-19 were subjected to chest CT as an auxiliary tool for the diagnosis. A radiologist evaluated CT scans by using the COVID-19 Reporting and Data System (CO-RADS). The scale is from 0 to 6, and each category represents the level of suspicion for pulmonary involvement in COVID-19. CO-RADS 0 was chosen if none of the categories from 1 to 5 could be assigned because scans were incomplete or of insufficient quality. CO-RADS 1 indicated findings with a very low level of suspicion (normal or non-infectious etiology). CO-RADS 2 indicated a low level of suspicion, with findings typical for infections other than COVID-19. CO-RADS 3 is an equivocal/unsure level of suspicion, representing features compatible with COVID-19, but also other diseases. CO-RADS 4 indicated a high level of suspicion for COVID-19. CO-RADS 5 was a very-high level of suspicion for pulmonary involvement, as is typical for COVID-19 findings; the mandatory features for CO-RADS 5 were ground-glass opacities with or without consolidations in lung regions close to visceral pleural surfaces, including the fissures and a multifocal bilateral distribution. Finally, CO-RADS 6 included those patients with a positive SARS-CoV-2 RT-PCR result.

### 2.5. SARS-CoV-2 RT-PCR Screening

At hospital admission, health workers identified patients with suspicion of COVID-19 based on clinical and/or radiological data, and a first RT-PCR was performed by using a nasopharynx or oropharynx swab. The first SARS-CoV-2 screening employed RT-PCR based on the Berlin protocol and was performed at the Health Public Laboratory of Zacatecas State. This laboratory is part of the National network of Viral Respiratory Disease Monitoring Health Units and is part of the Health Secretary of Zacatecas. If the first RT-PCR result was negative, a second nasopharynx swab was obtained and sent to the Molecular Medicine Laboratory from Academic Unit of Human Medicine and Health Sciences from the Autonomous University of Zacatecas Francisco García Salinas for viral screening. This laboratory has been authorized as a COVID-19 diagnosis laboratory by the Instituto de Diagnóstico y Referencia Epidemiológicos Dr. Manuel Martínez Báez, which is the institution of the Ministry of Health of Mexico in charge of diagnosis, control, referral, research, and technological development for the surveillance of epidemiological diseases [13]. Exudate samples were screened for this second time for SARS-CoV-2 with a one-step RT-PCR assay, using the TaqMan 2019nCoV assay (Thermo Fisher Scientific, Waltham, MA, USA) according to the manufacturer indications. This RT-PCR assay is based on detection of the SARS-CoV-2 genes N and S and the ORF1 region. Both laboratories that carried out the COVID-19 diagnosis for the protocol had 100% concordance in their results when they carried out a blinded independent COVID-19 diagnosis from the same panel of samples.

### 2.6. Statistical Analysis

The Shapiro–Wilk test was used to assess the normality of the variables. For normally distributed variables, Levene’s test was applied to check homoscedasticity. Student’s *t*-test was applied when the variances were homogenous. When the variances were not homogenous, Welch’s test was applied. For non-normally distributed variables, the Mann–Whitney–Wilcoxon test was applied for comparisons between study groups. Categorical variables were compared by using Fisher’s exact test or the chi-square test. The phi coefficient and Cramer’s V were used to measure the strength of association between a CO-RADS 5 chest CT and a positive second RT-PCR. Multivariate logistic regression was used to evaluate independent predictors of COVID-19 diagnosis during the second RT-PCR, with COVID-19 diagnosis as the dependent variable. Statistical analysis was performed with R Studio Cloud 4.0.3 (https://rstudio.cloud/project/2614648. Accessed on 6 January 2022), and *p* < 0.05 was considered statistically significant.

## 3. Results

During the period in which the study was carried out (November 2020 to May 2021), 709 patients were hospitalized with suspicion of respiratory viral infection and underwent a first SARS-CoV-2 screening. From these patients, 55 were negative, but they had clinical/radiological findings related to COVID-19 and therefore were included form the study. These patients underwent a second SARS-CoV-2 RT-PCR. Thirty-six (65.5%) of the participants were positive for SARS-CoV-2; these patients comprised the COVID-19 group. The remaining 19 (34.5%) patients were negative for SARS-CoV-2 and comprised the control group. Table 1 displays the characteristics of the general population and stratified into the COVID-19 and control groups.

The average age of the study population was 57.3 years; 31 (56.4%) patients were men. The presence of comorbidities at the time of inclusion in the study was as follows: 32.7% of patients had type 2 diabetes mellitus (T2DM), 40% had systemic arterial hypertension, and 34.5% presented obesity. At the time of admission, there were no differences between the study groups in age, sex, comorbidities, and symptoms (*p* > 0.05).

Considering the first SARS-CoV-2 screening and the beginning of symptoms, the mean COVID-19 testing time was 9.97 ± 5.10 days for the COVID-19 group and 10.84 ± 7.63 days for the control group (*p* = 0.94). The second SARS-CoV-2 screening was performed 13.72 ± 5.38 days after symptoms began for the COVID-19 group and 13.53 ± 7.43 days after symptoms began for the control group (*p* = 0.6). Considering the need for supplemental oxygen during hospitalization, 85.5% of patients used at least nasal cannulas with supplementary oxygen, up to 90.9% used a facemask, and 18.2% of patients required mechanical ventilation (Table 1). There were no differences in the type of supplemental oxygen used during the hospital stay between the COVID-19 and control groups (*p* > 0.05).

Table 2 displays the laboratory findings of the overall study population and for each study group. Considering the obtained data of hematic biometry, the platelet count differed between the COVID-19 and control groups (*p* = 0.01). The glucose levels were 167.58 ± 93.09 mg/dL and 124.16 ± 58.37 mg/dL in the COVID-19 and control groups, respectively (*p* = 0.03). The partial pressure of oxygen was lower in the COVID-19 group (68.50 ± 31.69 mmHg) than in the control group (81.42 ± 40.43 mmHg); this difference was significant (*p* = 0.04).

Regarding the biochemical parameters related to inflammation/prothrombotic events and compared with controls, D-dimer was slightly increased in the COVID-19 group, with an average value of 5.24 pg/mL (*p* = 0.003). There was a significant increase in procalcitonin of the control group (4.88 ± 14.26 pg/mL) compared with the COVID-19 group (0.84 ± 1.17 pg/mL) (*p* = 0.005). There were no differences in the other biochemical markers between the groups (*p* > 0.05).

**Figure 1:**
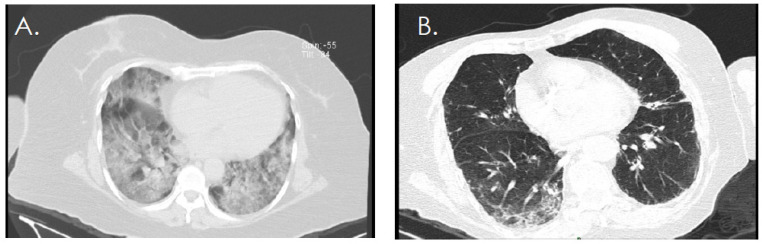
Representative chest-computed axial tomography (CT) slices. Each patient was screened based on anatomical abnormalities observed on chest CT scans. (**A**) Chest CT findings categorized as CO-RADS 5 and (**B**) chest CT findings classified as CO-RADS 3. CO-RADS, COVID-19 Reporting and Data System.

**Figure 2:**
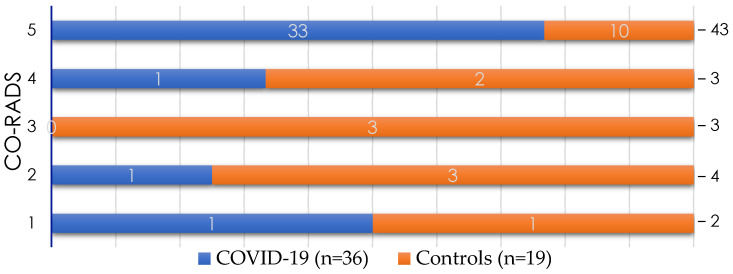
Chest-computed axial tomography (CT) findings according to the COVID-19 Reporting and Data System (CO-RADS) classification. The study population with a negative first SARS-CoV-2 RT-PCR result (*n* = 55) underwent a second SARS-CoV-2 RT-PCR. According to this result, they were classified into the COVID-19 group (positive RT-PCR result) or the control group (negative RT-PCR result). At the time of admission, a chest CT was carried out, and the scans were categorized by using the CO-RADS system. The graph shows the number of patients in each group based on their CO-RADS score. Most of the patients in the COVID-19 group were classified as CO-RADS 5.

Figure 1 and Figure 2 shows the results of the chest CT, evaluated by using the CO-RADS system. Of the study population, 78.18% of the patients presented CO-RADS 5. As expected, CO-RADS 5 findings were significantly more frequent in the COVID-19 group (91.66%) than in the control group (52.63%) (*p* = 0.003). In the COVID-19 group, only three patients had a CO-RADS of 1, 2, or 4 (Figure 2).

The phi coefficient and Cramer’s V were calculated to determine the strength of the association between reaching a specific CO-RADS score and having a positive second SARS-CoV-2 RT-PCR. The phi coefficient was 0.42, and Cramer’s V was 0.43. Considering the results of the second SARS-CoV-2 RT-PCR and chest CT classified as CO-RADS 5, 42 of 55 patients were correctly classified, a concordance of 76.36% between the methods.

After statistical modeling by multiple logistic regression and considering SARS-CoV-2 infection as the dependent variable, only radiological findings classified as CO-RADS 5 remained an independent predictor of COVID-19 for a second viral screening (odds ratio = 7.0, 95%; confidence interval, 1.5–32.7; *p* = 0.013).

## 4. Discussion

The timely and accurate diagnosis of COVID-19 in hospitalized patients is important to prevent the spread of the virus and to provide the patient with the correct treatment. Considering that there are reports of patients with a high clinical suspicion of COVID-19 but with negative SARS-CoV-2 RT-PCR, which demonstrates the presence of false negatives in COVID-19 molecular diagnostic methods, we evaluated potential false-negative results in hospitalized patients suspected of viral respiratory disease but with a negative previous SARS-CoV-2 RT-PCR result. We also evaluated variables that may increase the success of COVID-19 diagnosis in this group of patients.

In our study, the most frequent comorbidities in hospitalized patients were hypertension (40%), obesity (34.5%), and T2DM (32.7%). These findings are in agreement with other studies [24,25]. However, there were no differences between the COVID-19 and control groups in the proportions of these comorbidities, age, or sex. These data demonstrate that the presence of comorbidities and risk factors may worsen an infectious disease (including COVID-19), but they were not associated with a false-negative diagnostic-test result among the study population. Moreover, we did not observe differences in the proportions of symptoms between study groups. This result is not unexpected, considering that, in Mexico, a “COVID-19-suspicious patient” needs to fulfil the criteria of the operational definition for respiratory infection [26], and our participants fit this definition. The most prevalent symptoms were dyspnea, fever, and asthenia. Of note, even though there was no significant difference between the COVID-19 and control group, no patient with two negative SARS-CoV-2 RT-PCR results presented anosmia, which has been described as a characteristic symptom of COVID-19, mainly in mild illness or illness with few symptoms [27].

Our univariant analysis of biochemical parameters showed that both the platelet count and D-dimer levels were higher in the COVID-19 group compared with the control group. The increased platelet number and D-dimer levels correlate with COVID-19-associated coagulopathy, which includes platelet activation and leads to increased inflammation (involving monocytes, dendritic cells, neutrophils, and activated T cells, and proteases of coagulation) [28]. D-dimer, a product of fibrin degradation, correlates with the activity of tissue plasminogen activator (t-PA), plasmin, and other fibrinolytic pathways [29]. These results indicate a relationship between proinflammatory conditions of patients that is caused by COVID-19, and it is been associated with the gravity of the infection [28,29]. Procalcitonin is a glycoprotein precursor of calcitonin; it does not have hormonal activity, and its levels are usually undetectable, but it can increase with bacterial infections; hence, it has been proposed as a biomarker to distinguish between bacterial and viral infections [11]. In our study, the COVID-19 group presented lower procalcitonin levels compared with the control group; this could indicate the infectious etiology in the control group. The COVID-19 group showed a higher initial level of serum glucose and a decrease in the oxygen partial pressure compared with the control group. It is important to note that 32.7% of the patients had T2DM, but up to 78% of those patients had an altered glucose value, perhaps due to stress from the illness per se and possibly related to increased metabolism for viral replication. Compared with the control group, the oxygen partial pressure was lower in the COVID-19 group. This phenomenon may be explained because, in moderate and severe COVID-19, hypoxemic respiratory insufficiency due to diffuse alveolar damage could lead to acute respiratory syndrome, as is consistent with the Berlin criteria [30].

In our study, a positive second SARS-CoV-2 RT-PCR result after a negative first test improved the COVID-19 diagnosis by 65%. In this context, the chest CT at the time of hospital admission classified as CO-RADS 5 was a predictive factor for a positive second SARS-CoV-2 RT-PCR result. There was a strong correlation between these variables, with Cramer’s V of 0.43 and a phi correlation coefficient of 0.42. A chest CT result classified as CO-RADS-5 increased the odds of obtaining a positive result from a second SARS-CoV-2 RT-PCR by seven times in the studied population. The relationship between CT findings and a positive result in the second SARS-CoV-2 RT-PCR had already been addressed [31,32,33,34]. As an example Xie et al. evaluated five patients with a negative RT-PCR result but with a high suspicion of SARS-CoV-2 infection with the aim of describing radiologic findings based on an initial CT scan. Three of their patients who had a chest CT scan highly compatible with COVID-19 pneumonia and a negative first SARS-CoV-2 RT-PCR result were diagnosed with COVID-19 after a second screening carried out 2–8 days after the first screening [31]. In contrast to our study—where we used the CO-RADS scale—those authors proposed a scale with a maximum of 24 points, considering specific areas and the severity of the damage. Besides evaluating findings, the CO-RADS scale [35] correlates with the severity of the infection, has high interobserver correlation, and the false-positive rate for CO-RADS 5 has been reported to be as low as 0–3%, making chest CT a great tool in COVID-19 diagnostics after a negative initial RT-PCR result. Based on our data, CT findings compatible with COVID-19 in combination with a second SARS-CoV-2 RT-PCR that is positive could increase the percentage of COVID-19 diagnosis by up to 65.5%. In our study, despite the differences in biochemical variables between the COVID-19 and control groups—platelet count and D-dimer, procalcitonin, and/or glucose levels—they did not have an independent predictive value for COVID-19 diagnosis if a patient underwent a second SARS-CoV-2 RT-PCR after a false-negative result from the first RT-PCR. After progress in vaccination coverage and the changes in genetic variability of the virus, most of the COVID-19 cases are mild. Therefore, the use of imaging results remains as a very useful tool in moderate, severe, or unclear cases.

Considering the 709 patients hospitalized with suspicion of respiratory viral infection who underwent a first SARS-CoV-2 screening during the period in which the study was carried out, the false-negative rate of RT-PCR was 5%. A false-negative result could have several causes, including the sampling technique, sample storage, sample transportation and processing, and the correct time of sampling relative to the beginning of the symptoms. Sensitivity and specificity can be influenced by the amplification protocols and the detected target. A false-negative result during the first screening may also be because the screening protocol includes only one SARS-CoV-2 gene (RdRp, according to the guidelines of the Mexican health institutions). The second SARS-CoV-2 RT-PCR is more specific for the virus—the target genes are N and S, as well as the ORF1 region of SARS-CoV-2—and this difference may increase the accuracy of detection. Another factor to consider is that we selected our population based on the criteria for the severity of the infection, because, in Mexico, only patients with moderate, severe, and critical illness are hospitalized. Hence, the higher replication and viral count are probably at the bottom of the airway, so the sampling location might not have contained the minimum viral quantity required for detection. Even though RT-PCR kits are able to detect each SARS-CoV-2 variant, the Omicron variant has demonstrated significantly lower replication in lower airway in comparison to the Delta variant, which was the most prevalent SARS-CoV-2 variant in Mexico at the time the study was realized [36]. The average 3-day difference between the first and second SARS-CoV-2 test is due to time required to receive results from the first laboratory. This delay may also influence the differences in successful SARS-CoV-2 detection.

## 5. Conclusions

We showed that, in hospitalized patients with a negative first SARS-CoV-2 RT-PCR result but with clinical findings related to COVID-19, the platelet count; glucose, D-dimer, and procalcitonin levels; and/or a chest CT classified as CO-RADS 5 differed between the COVID-19 and control groups. However, only the chest CT classified as CO-RADS 5 was an independent predictor of a positive second SARS-CoV-2 RT-PCR, increasing the odds of COVID-19 diagnosis by seven times. Based on our results, in hospitalized patients with a chest CT classified as CO-RADS 5, a second SARS-CoV-2 RT-PCR test should be mandatory when the first one is negative. This approach could increase SARS-CoV-2 detection by up to 65.5% and could allow for the isolation of these patients and provision of correct treatment, improving the patient outcome and avoiding further contagion.

## Figures and Tables

**Table 1:** General data of the study population classified into the COVID-19 and control groups according to the second SARS-CoV-2 RT-PCR.

Variable	General Population (*n* = 55)	Study Group		*p*-Value
		COVID-19 (*n* = 36)	Controls (*n* = 19)	
Age (years)	57.3 ± 16.77	57.94 ± 15.94	56.11 ± 18.64	0.69
Sex female	24 (43.6)	14 (38.8)	10 (52.6)	0.39
Comorbidities (*n*, %)				
Hypertension	22 (40)	12 (33.3)	10 (52.6)	0.17
Diabetes	18 (32.72)	13 (36.1)	5 (26.3)	0.46
Obesity	19 (34.54)	13 (36.1)	6 (66.6)	0.74
Other comorbidity	26 (47.27)	15 (41.7)	11 (57.8)	0.25
Symptoms * (*n*, %)				
Cough	33 (60)	21 (58)	12 (63)	0.73
Dyspnoea	53 (96.36)	36 (100)	17 (89)	0.12
Chest pain	14 (25.45)	10 (27.7)	4 (21)	0.75
Fever	38 (69.09)	26 (72.2)	12 (63.1)	0.49
Anosmia	4 (7.27)	4 (11.1)	0	0.29
Dysgeusia	5 (9.09)	4 (11)	1 (5.2)	0.65
Asthenia	34 (61.1)	25 (69.4)	9 (47.3)	0.11
Myalgias	16 (29.09)	11 (39.5)	5 (26.3)	0.74
Arthralgias	16 (29.09)	13 (36.1)	3 (15.7)	0.12
Diarrhea	8 (14.54)	4 (11)	4 (21)	0.43
Headache	14 (25.45)	11 (30.5)	3 (15.7)	0.33
Sickness	12 (21.81)	9 (25)	3 (15.7)	0.51
Supplementary oxygen ** (*n*, %)				
Nasal cannula	47 (85.45)	30 (83.3)	17 (89.5)	0.7
Facemask	50 (90.90)	33 (91.7)	17 (89.5)	0.78
Mechanic Ventilation	10 (18.18)	6 (16.7)	4 (21.1)	0.72
COVID-19 testing moment (days)				
First COVID-19 test	10.27 ± 36.46	9.97 ± 5.10	10.84 ± 7.63	0.94
Second COVID-19 test	13.65 ± 6.09	13.72 ± 5.38	13.53 ± 7.43	0.6
Cq value (mean ± SD)				
ORF1 gene	-	29.24 ± 4.44	-	-
N gene	-	28.52 ± 5.08	-	-
S gene	-	29.17 ± 4.31	-	-

* The reported symptoms are from the time of the hospital admission. ** Supplemental oxygen needed during hospitalization.

**Table 2:** Laboratory findings of the study groups at the time of hospital admission.

Laboratory Finding	General Population (*n* = 55)	Study Group		*p*-Value
		COVID-19 (*n* = 36)	Controls (*n* = 19)	
Hematic biometry (mean ± SD)				
Hemoglobin (g/dL)	13.54 ± 3.11	13.97 ± 2.73	12.73 ± 3.69	0.20
Platelets (10^3^/dL)	267.4 ± 117.7	296.5 ± 105.8	212.4 ± 122.1	0.01 *
Leucocytes (counts/dL)	10,733 ± 4853	10,797 ± 4608	10,611 ± 5418	0.85
Lymphocytes (counts/dL)	1069 ± 620	1147 ± 711	922 ± 367	0.29
Neutrophils (counts/dL)	9261 ± 4462	9347 ± 4175	9098 ± 5079	0.85
Blood chemistry (mean ± SD)				
Prothrombin time (s)	16.08 ± 2.77	15.77 ± 2.4	16.63 ± 3.35	0.33
Partial thromboplastin time (s)	36.80 ± 11.10	36.1 ± 11.6	38.06 ± 10.36	0.26
Glucose (mg/dL)	152.58 ± 84.77	167.58 ± 93.09	124.16 ± 58.37	0.03 *
Creatinine (mg/dL)	1.91 ± 4.67	1.99 ± 5.53	1.76 ± 2.44	0.42
Blood urea nitrogen (mg/dL)	28.15 ± 22.71	27.97 ± 25.09	28.511± 7.99	0.37
Direct bilirrubin (mg/dL)	0.10 ± 0.79	0.34 ± 0.23	0.82 ±1.29	0.15
Albumin (mg/dL)	3.10 ± 0.48	3.12 ± 0.47	3.07 ± 0.51	0.75
Arterial blood gas test (mean ± SD)				
pH	7.42 ± 0.08	7.43 ± 0.06	7.41 ± 0.10	0.61
pO_2_ (mmHg)	72.96 ± 35.13	68.50 ± 31.69	81.42 ± 40.43	0.04 *
pCO_2_ (mmHg)	33.13 ± 8.95	32.39 ±7.47	34.53 ± 11.34	0.51
HCO_3_ (mmol/L)	22.22 ± 3.96	22.29 ± 4.30	22.08 ± 3.3	0.84
Inflammatory markers (mean ± SD)				
Fibrinogen (mg/dL)	573.97 ± 193.28	615.91± 186.34	512.47 ± 192.77	0.84
D-dimer (pg/mL)	4.07 ± 6.21	5.24 ± 7.42	3.23 ± 5.28	0.003 *
Lactate dehydrogenase (mg/dL)	639.03 ± 402.47	620.79 ± 405.07	675.53 ± 407.02	0.89
Ferritin (ng/mL)	765.93 ± 744.26	692.85 ± 576.70	912.52 ± 1019.16	0.41
Procalcitonin (mg/dL)	2.41 ± 8.96	0.84 ± 1.17	4.88 ± 14.26	0.005 *
C-reactive protein	16.55 ± 11.62	16.26 ± 9.32	17.05 ± 15.06	0.73
Troponin (ng/mL)	1.03 ± 4.15	0.31± 0.44	1.81 ± 5.98	0.42
Creatine kinase (mg/dL)	413.08 ± 1472	567.41 ± 1922.82	200.9 ± 295.2	0.56

The data are presented as the mean ± standard deviation; * *p* < 0.05. pH, hydrogen potential; pO_2_, partial pressure of oxygen; pCO_2_, partial pressure of carbon dioxide; HCO_3_, bicarbonate.

## Data Availability

Data that support the findings of this study are available from the corresponding authors (M.L.M.-F. and I.G.-V.) upon reasonable request.
